# Evaluating joint-space narrowing and cartilage loss in rheumatoid arthritis by using MRI

**DOI:** 10.1186/ar3861

**Published:** 2012-05-30

**Authors:** Charles G Peterfy, Julie C DiCarlo, Ewa Olech, Maire-Agnes Bagnard, Annarita Gabriele, Norman Gaylis

**Affiliations:** 1 Spire Sciences, LLC, 72 Rock Road, San Francisco, CA 94904, USA; 2 Department of Rheumatology, University of Nevada School of Medicine, 1707 West Charleston Boulevard, Suite 220, Las Vegas, NV 89102, USA; 3 F. Hoffman-La Roche AG, Konzern-Hauptsitz, Grenzacherstrasse 124, CH-4070 Basel, Switzerland; 4 Arthritis & Rheumatic Disease Specialties, 21097 NE 27th Court, Aventura, FL 33180, USA

## Abstract

**Introduction:**

Magnetic resonance imaging (MRI) has been shown to be superior to radiography (XR) for assessing synovitis, osteitis, and bone erosion in rheumatoid arthritis (RA), particularly in clinical trials. However, relatively little has been reported on the ability of MRI to evaluate articular cartilage loss, or joint-space narrowing (JSN), in the hands and wrists. In a previous study, we adapted the nine-point Genant-modified Sharp XR-JSN score for use with MRI (MRI-JSN). In this study, we compare MRI-JSN with XR-JSN by using images from two multicenter clinical trials.

**Methods:**

Baseline XR and 1.5-Tesla MR images of one hand and wrist from each of 47 subjects with RA enrolled in one of two multicenter clinical trials were evaluated by using the XR-JSN and MRI-JSN methods by a single radiologist experienced in the two methods. Radiographs and MR images were read independently on different occasions.

**Results:**

In total, 575 of 611 joints were compared (one metacarpophalangeal joint of the thumb and 35 proximal interphalangeal joints were outside the MRI field of view and could not be assessed). The 22 (47%) subjects showed JSN with both XR and MRI, and 25 (53%) subjects showed no JSN with either method. No subject showed JSN with only one or the other method. MRI showed high agreement with XR (intraclass correlation coefficient = 0.83). Sensitivity of MRI for JSN, by using XR as the gold standard, was 0.94; specificity was 0.91; accuracy was 0.91; positive predictive value was 0.64; and negative predictive value was 0.99.

**Conclusions:**

This validation exercise suggests that MRI JSN scoring may offer a viable alternative to XR JSN scoring in multicenter clinical trials of RA. However, the relative longitudinal sensitivity of MRI to change and the ability to discriminate therapeutic effect on JSN were not evaluated in this study.

## Introduction

The past decade has seen remarkable advances in structure-modifying treatment of rheumatoid arthritis (RA), with several effective therapies having been shown to slow or stop the progression of radiographic (XR) bone erosion and joint-space narrowing. However, many patients do not respond fully to these therapies, or experience side effects, so further development is still needed. One obstacle to progress in this regard is that clinical trials of putative new therapies are becoming harder to perform with XR. This is in part because the introduction of effective therapies has made pure placebo-controlled studies no longer ethical, necessitating a shift to add-on and active-comparator study designs [1], which exhibit slower progression and smaller differences between treatment arms, and therefore require longer observation periods and larger numbers of patients to achieve discriminative power. Additionally, early rescue treatment for patients showing poor response complicates interpretation of long-term study results and makes detecting structural change quickly even more important. These factors, along with the decreasing availability of RA patients appropriate for such studies, have increased the cost of clinical trials and threaten to impede progress in therapeutic development.

MRI has been shown to be more sensitive than XR for detecting bone erosion in patients with RA [2-9] and thus offers a potential solution to this problem. However, relatively little has been reported on the ability of MRI to evaluate cartilage loss or JSN in the hands and wrists of RA patients [4,8,10]. In a previous study [7], we adapted the Genant-modified Sharp XR scoring method [11] for use with MRI in the hand, wrist, and foot, and found MRI to have greater sensitivity to change for both bone erosion and JSN than did XR [7]. We and others have also shown MRI adaptations of the 5-point van der Heijde-Sharp XR-JSN score to be effective [4,8,10]. In this study, we examined the diagnostic accuracy of the 9-point MRI-JSN score [7] based on the corresponding 9-point XR-JSN score as a gold standard, by using images acquired from two multicenter clinical trials.

## Materials and methods

In total, 47 subjects (77% women, mean age 46 years) with RA enrolled in one of two multicenter randomized, controlled trials (24 patients from IMPRESS (Impact of Rituximab on Magnetic Resonance Imaging Evidence of Synovitis and Bone Lesions in Patients With Moderate or Severe Rheumatoid Arthritis) and 23 patients from RA SCORE (A Study of MabThera (Rituximab) in Patients with Rheumatoid Arthritis and Inadequate Response to Methotrexate)) were included in this evaluation. All subjects had baseline XR and MRI of one hand and wrist by using standardized image-acquisition protocols, as described later. Both study protocols underwent institutional board review and received ethical approval, and all patients provided informed consent to participate in the studies.

### MRI

One hand and wrist of each patient was imaged with a 1.5-T whole-body MRI scanner by using a commercial surface coil. Reproducible positioning was ensured with a specially designed acrylic hand frame. Pulse sequences included coronal, T_1_-weighted, three-dimensional (3D) gradient-echo with spectral fat suppression. Repetition time (TR) was 43 milliseconds, echo time (TE) was 12 milliseconds, the field of view was 120 mm, the matrix was 512 × 195, and the slice thickness was 1.5 mm, giving a voxel resolution of 234 μm × 625 μm × 1,500 μm. Only one excitation was averaged. Anatomic coverage extended from the distal radioulnar joint proximally to the proximal interphalangeal (PIP) joints distally, and included the entire thumb. The joints of the hands were scanned separately from those of the wrist.

### Radiography

Each of the two hands/wrists and feet of every patient were radiographed separately on high-resolution 10-inch × 12 inch single-emulsion, single-screen film, by using standardized positioning with a template. Hands/wrists were exposed postero-anteriorly, with the x-ray beam centered between the second and third metacarpophalangeal (MCP) joints and perpendicular to the cassette. Radiographs were digitized to a pixel size of 100 microns at 12-bits per pixel.

### Image analysis

**Figure 1:**
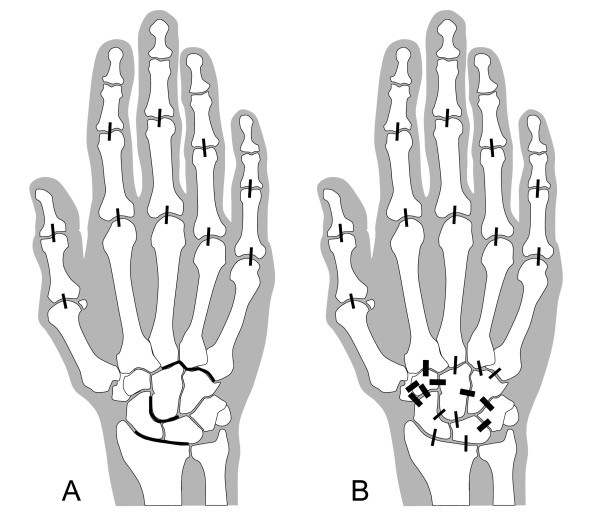
**Locations evaluated for JSN**. XR-JSN **(A) **was scored in 13 locations as per the Genant-modified Sharp method (thin lines: proximal interphalangeal (PIP) joints 1 to 5, metacarpophalangeal (MCP) joints 1 to 5, carpometacarpal (CMC) joints 3 to 5 as a unit, capitate-lunate-scaphoid joints as a unit, and scaphoid-radius and lunate-radius joints as a unit). MRI-JSN **(B) **was scored in these same 17 joints (thin lines) plus eight additional joints (thick lines: trapezoid-trapezium joint, scaphoid-trapezium joint, scaphoid-trapezoid joint, CMC joint 2, capitate-trapezoid joint, capitate-hamate joint, hamate-triquetrum joint, and triquetrum-lunate joint).

All radiographs were assessed for JSN by using the Genant-modified Sharp method, in which 13 locations involving 17 joints (interphalangeal (IP), 1; PIP, 2; PIP, 3; PIP, 4; PIP, 5; MCP, 1; MCP, 2; MCP, 3; MCP, 4; MCP, 5; carpometacarpal (CMC), 3 to 5 as a unit; capitate-lunate-scaphoid space as a unit; and radiocarpal space (scaphoid-radius and lunate-radius joints as a unit)) (Figure 1a) in each hand/wrist were scored on a 9-point scale ranging from 0 to 4 in increments of 0.5 [11]. The maximum XR-JSN score per patient was 52. All MR images were assessed for JSN by using the same 9-point scale (0.0, no cartilage loss or JSN; 0.5, equivocal cartilage loss or JSN; 1.0, minimal (< 10%) but definitive cartilage loss or JSN; 1.5, mild (10% to 25%) cartilage loss or JSN; 2.0, moderate cartilage loss or JSN (26% to 75%, including unilaterally denuded areas but no bilaterally denuded areas or bone-on-bone contact); 2.5, moderate-severe cartilage loss or JSN (> 75%, including focal denuding or focal bone-on-bone contact); 3.0, complete cartilage denuding or diffuse bone-on-bone contact; 3.5, partial ankylosis; 4.0, complete ankylosis) in 25 joints (IP 1, PIP 2, PIP 3, PIP 4, PIP 5, MCP 1, MCP 2, MCP 3, MCP 4, MCP 5, CMC 2, CMC 3, CMC 4, CMC 5, hamate-capitate, hamate-triquetrum, triquetrum-lunate, capitate-lunate, capitate-scaphoid, capitate-trapezoid, trapezoid-trapezium, scaphoid-trapezium, scaphoid-trapezoid, radius-scaphoid, radius-lunate) (Figure 1b) in each hand/wrist. Carpometacarpal joint 1 was excluded because of the high frequency of osteoarthritic JSN in this location. The scapholunate joint was excluded because rupture of the scapholunate ligament often widens this joint. The triquetropisiform joint was excluded because it is not well visualized in the coronal plane. The distal radioulnar joint was excluded because it is not load bearing and because of difficulty in reproducibly aligning the joint on serial MRI examinations. Additionally, this joint was found in previous radiographic studies to be among the least frequently involved locations in the hand and wrist [12].

The maximum MRI-JSN score was 100. Radiographs and MR images were read independently, in random order, and on different occasions by a single radiologist (CP) experienced in XR and MRI evaluation of RA in clinical trials and blinded to patient identifiers.

MRI-JSN scores were compared with XR-JSN scores for the 13 joints included in the Genant-modified Sharp method. For the purposes of this comparison, the highest MRI-JSN score among the capitate-lunate and capitate-scaphoid joints was used to correspond to the capitate-lunate-scaphoid space scored with XR-JSN. Similarly, the highest MRI-JSN score between the radius-scaphoid and radius-lunate joints was used to correspond to the radiocarpal joint scored with XR-JSN. Agreement between XR-JSN and MRI-JSN scores was determined by intraclass correlation coefficient (ICC). Sensitivity (true positive/(true positive + false negative)), specificity (true negative/(true negative + false positive)), accuracy ((true positive + true negative)/(true positive + true negative + false positive + false negative)), positive predictive value (true positive/(true positive + false positive)) and negative predictive value (true negative/(true negative + false negative)) of MRI-JSN were calculated by using XR-JSN as the gold standard.

Receiver operating characteristic (ROC) curve analysis was also performed by using XR-JSN as the gold standard.

## Results

**Figure 2:**
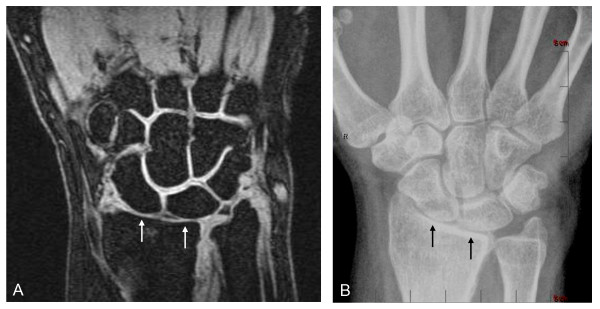
**JSN depiction in wrist joints**. Coronal fat-suppressed T_1_-weighted gradient-echo image of wrist **(A) **shows sharp delineation of bone margins free of chemical-shift artifacts, thus allowing accurate determination of the joint-space widths, corresponding closely to those seen with XR in the same wrist **(B)**. Note how clearly both MRI and XR show the radius-lunate and radius-scaphoid joint spaces (arrows) to be narrowed (JSN) relative to the other joints in the field of view. A JSN score of 2.0 was given to these joints independently on MRI and XR. MRI **(A) **additionally depicts the articular cartilage directly as high-signal tissue lining the articular cortices of the bones and showing sharp contrast with adjacent low-signal joint fluid on one side and low-signal articular bone and subcortical marrow fat on the other. As illustrated in this example, the interfaces between opposing cartilage surfaces, particularly in normal joints, often can be sharply delineated. Note that although the radius-lunate and radius-scaphoid joints are clearly narrowed on both MRI and XR, MRI further shows the cartilage to be only thinned on both sides of these joints, without complete denuding in any location. This important distinction cannot be determined with XR.

**Figure 3:**
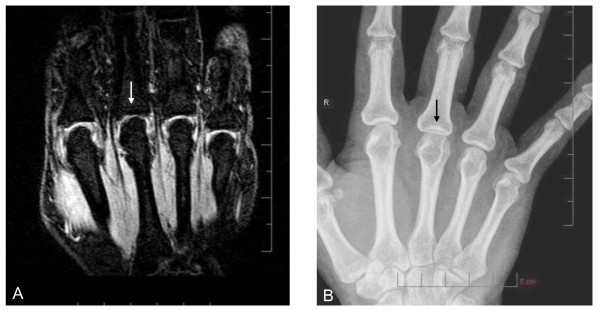
**Joint-space narrowing (JSN) depiction in **metacarpophalangeal **(MCP) joint**. Coronal, fat-suppressed, T_1_-weighted gradient-echo image of MCP joints 2 through 5 **(A)**, and corresponding XR image **(B) **show grade-2.0 JSN of MCP 3 (arrow) but normal joint-space width of MCP 2, 4, and 5. The proximal interphalangeal joints are out of the plane of section on the MR image shown, but were visible on more-palmar sections of the scan (not shown).

**Figure 4:**
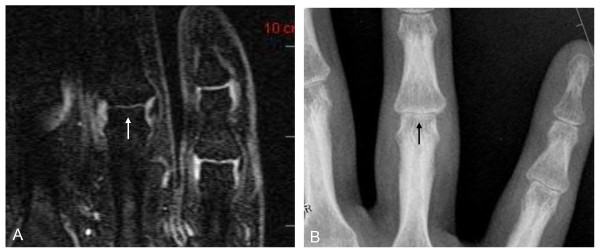
**Joint-space narrowing (JSN) depiction in proximal interphalangeal (PIP) joint**. Coronal, fat-suppressed, T_1_-weighted gradient-echo image **(A) **and XR **(B) **show grade 2 JSN of proximal interphalangeal joint 4 (arrow).

In total, 575 of 611 joints were compared (one MCP joint of the thumb and 35 proximal interphalangeal joints were outside the MRI field of view and could not be assessed). Twenty-two (47%) subjects showed JSN with both XR and MRI. Figures 2, 3, and 4 show examples of such cases. Twenty-five (53%) subjects showed no JSN with either method. MRI detected 44 more joints with JSN than XR did, but missed two PIP joints with JSN on XR. Mean MRI-JSN score was 6.7 (SD, 11.7) for all 25 joints and 4.2 (SD, 6.4) for the 13 joints also evaluated with XR. Mean XR-JSN score for these 13 joints was 3.0 (SD, 5.3). No statistically significant difference was found between MRI-JSN and XR-JSN scores for these 13 joints.

Table 1 summarizes the findings on a per-joint basis. As can be seen, 89% of joints showed identical scores with MRI as with XR. MRI-JSN also showed high agreement with XR-JSN based on ICC (0.83).

**Table 1:** Number and percentage of joints showing correlation of MRI-joint-space narrowing (JSN) with XR-JSN

	MRI-JSN score									
XR-JSN score	0.0	0.5	1.0	1.5	2.0	2.5	3.0	3.5	4.0	Total
0.0	**449 (78.1%**)	0	32 (5.6%)	0	12 (2.1%)	0	0	0	0	493 (85.7%)
0.5	0	**0**	0	0	0	0	0	0	0	0
1.0	2 (0.3%)	0	**22 (3.8%)**	0	5 (0.9%)	0	0	0	0	29 (5.4%)
1.5	0	0	1 (0.2%)	**0**	1 (0.2%)	0	0	0	0	2 (0.3%)
2.0	0	0	1 (0.2%)	0	**30 (5.2%)**	7 (1.2%)	0	0	0	38 (6.6%)
2.5	0	0	0	0	0	**7 (1.2%)**	1 (0.2%)	1 (0.2%)	0	9 (1.6%)
3.0	0	0	0	0	0	0	**3 (0.5%)**	1 (0.2%)	0	4 (0.7%)
3.5	0	0	0	0	0	0	0	**0**	0	0
4.0	0	0	0	0	0	0	0	0	**0**	0
Totals	451 (78.4%)	0	56 (9.7%)	0	48 (8.3%)	14 (2.4%)	6 (1.0%)	2 (0.3%)	0	575 (100%)

**Bold **values indicate exact agreement between 9-point Genant-modified Sharp XR-JSN score MRI-JSN and XR-JSN.

**Figure 5:**
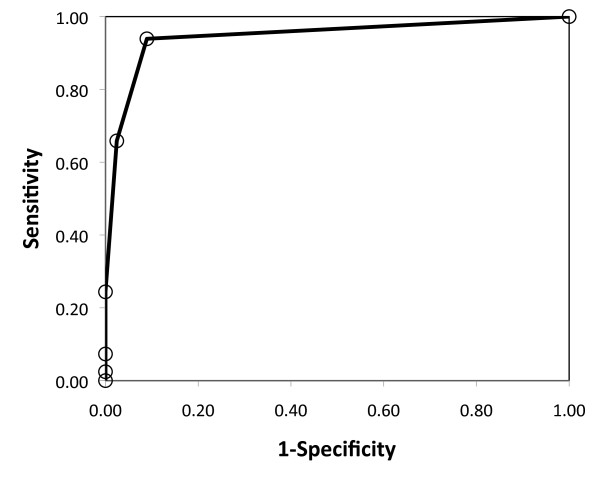
**Receiver operating characteristic (ROC)**. The ROC curve shows relation between true-positive rate (sensitivity) and false-positive rate (1-specificity) of MRI-JSN based on XR-JSN as the gold standard, as the MRI-JSN positivity criterion is increased from 0.0 to 4.0. The prominent shift of the curve to the left of the no-discrimination line (broken line) is indicative of the high discriminative power of MRI for JSN.

Sensitivity of MRI for JSN, by using XR as the gold standard, was 0.94; specificity was 0.91; accuracy was 0.91; positive predictive value was 0.64; and negative predictive value was 0.99. ROC curve analysis (Figure 5) showed MRI to be highly discriminative of XR JSN, as defined by the Genant-modified Sharp score. The number of JSN-positive patients did not increase when XR-JSN scores were added from the other hand and both feet, which had not been imaged with MRI, but which typically are included in clinical trials using XR. However, when eight additional hand/wrist joints assessed with MRI were considered, a total of 36 JSN-positive joints and one JSN-positive patient were added.

## Discussion

In this validation exercise, MRI assessment of JSN in the hands and wrists of patients with RA provided the same results as did XR assessment of the same joints. MRI was both highly sensitive and specific for JSN that was demonstrable with XR. This suggests that MRI-JSN scoring may offer a viable alternative to XR-JSN scoring in multicenter clinical trials of RA. This is important because clinical trials with XR have become more costly and time consuming over the past decade. RA patients appropriate for clinical trials are increasingly difficult to recruit, and the switch to active comparator study designs has increased the number of patients and the observation time required to discriminate reliably the differences in change between treatment arms. The increased sensitivity of MRI for detecting bone erosions [2-9] should allow it to demonstrate structure-modifying treatment more quickly and with fewer patients than XR could. However, the MRI scoring method that has been used in most clinical trials thus far, the Outcome Measures in Rheumatology Clinical Trials RA MRI Score (OMERACT-RAMRIS) [13], did not include cartilage loss or JSN, and thus lacked the content validity of XR for structural joint damage. This was a significant limitation because bone erosion and cartilage loss do not always show the same pattern of response to therapy, as illustrated in the randomized, controlled trial of the inhibitor of the receptor for activated nuclear factor-κB (RANKL), denosumab, reported by Cohen *et al. *[9]. In that study, 227 patients with established RA were treated with either placebo plus methotrexate or one of two doses of denosumab plus methotrexate and followed up longitudinally with XR (erosion and JSN) and MRI (erosion only). Although both MRI and XR showed denosumab to have strong erosion-suppressing effects, XR showed denosumab to have no effect on preventing JSN. Had XR been excluded from the study, the lack of efficacy on this structural end point would not have been noticed.

Fortunately, MRI is well suited for imaging articular cartilage [14-18]. It depicts joint anatomy tomographically and therefore without projectional distortions that can mimic JSN on conventional XR, and MRI also is able to visualize the articular cartilage tissue directly, rather than only as a space between opposing articular cortices, as with XR. Moreover, MRI shows the same distribution of involvement of joints in the hands and wrists of patients with RA as does XR [19]. Fat-suppressed, T_1_-weighted 3D gradient-echo scans, as were used in this study, have been shown to delineate articular cartilage accurately in various joints, including the MCPs [15], and is commercially available on all clinical MRI systems operating at a magnetic field strength of 1.0 T or higher. Systems operating at lower field strengths currently have difficulty with this technique because of limitations in spectral fat suppression or selective water excitation. Selective fat suppression or water excitation are important for increasing T_1 _contrast between cartilage and adjacent joint fluid or subchondral bone (marrow fat) and for eliminating chemical-shift effects [16], which distort cartilage-bone interfaces and can simulate cartilage thinning and JSN. Increasing receiver bandwidth can reduce chemical shift, but this reduces the signal-to-noise ratio of the images and does not completely eliminate the problem. Fat-suppressed, T_1_-weighted, 3D gradient-echo is also the most commonly used pulse sequence for evaluating bone erosion in multicenter randomized controlled trials of RA [9,20-23]. Thus, MRI protocols do not require expansion to add JSN to assessments of joint damage.

One technical challenge in MRI-JSN assessment is achieving adequate coverage of all PIP joints, as these joints are at the distal limit of the field of view used in most clinical-trial protocols. In this study, 35 (19%) of 188 of PIP joints were not adequately covered and therefore excluded from analysis. Despite this limitation and the fact that images from two different multicenter clinical trials were pooled for this investigation, MRI-JSN correlated strongly with XR-JSN, attesting to the robustness of the MRI-JSN method.

Because of the longer imaging time required for MRI than for XR, MRI protocols in most clinical trials include only one or two hand(s)/wrist(s) per patient, whereas XR protocols typically include both hands/wrists and feet. Despite this greater anatomic coverage of XR, however, adding XR data from the other hand/wrist and both feet did not increase the number of JSN-positive patients in this study. Conversely, adding data from the eight additional joints included by MRI increased the number of JSN-positive joints by 36 and the number of JSN-positive patients by one. Thus, MRI of one hand/wrist seems to offer at least the same sensitivity for detecting RA patients with JSN as does XR of both hands and feet.

The MRI-JSN score used this study was modeled after the Genant-modified Sharp XR-JSN score [11], which has been used in multiple clinical trials to gain regulatory approval of structure-modifying therapies, including abatacept [24], rituximab [25], and tocilizumab [26]. In contrast to the van der Heijde-Sharp JSN-XR score, which uses a 5-point scale, ranging from 0 to 4 in increments of 1 [27], the Genant-modified Sharp XR-JSN score uses a 9-point scale, also ranging from 0 to 4, but in increments of 0.5. Both methods examine PIP 2, PIP 3, PIP 4, PIP 5, MCP 1, MCP 2, MCP 3, MCP 4, MCP 5, CMC 3, CMC 4, CMC 5, and the capitate-scaphoid and radius-scaphoid joints, but the Genant-modified Sharp method combines CMC 3 to 5 into a single space and adds the IP 1, capitate-lunate, and radius-lunate joints. Direct comparisons of these two XR scoring methods by using images from multicenter clinical trials, found that despite the differences in scales and specific joints examined by each, both methods showed the same discriminative power for XR-JSN change over time and between treatment arms [28,29]

The advantage of a 9-point scale over a 5-point scale is that including smaller increments across a similar range of structural damage may improve sensitivity to change, although comparative longitudinal studies are needed to evaluate this directly. Additionally, all images in this study were evaluated by a single, expert radiologist with 20 years of experience reading XR and MR images in RA clinical trials. As such, the results represent a best-case scenario and may not generalize to analyses performed by less-experienced readers. Interreader variability and smallest detectable change were not evaluated. Time required to perform MRI-JSN scoring also was not evaluated; however, because the technique is extremely detailed, it may be time consuming for inexperienced readers to perform. In a previous investigation [7], we demonstrated the 9-point MRI-JSN scale to be more sensitive to change than was the 9-point XR-JSN scale. We and others similarly found high interreader agreement and strong XR correlation of a 5-point MRI-JSN score that was similar to the van der Heijde XR-JSN score [4,8,10]. McQueen *et al. *[4] recently compared MRI with XR by using such a 5-point scale in the wrists of 38 patients with RA and 22 control subjects by using 2D spin-echo and 3D gradient-echo MRI at 3 T but without fat suppression, except on postcontrast 3D scans. Although chemical-shift artifacts are greater at 3 T than at 1.5 T, and the joints evaluated by MRI and XR in their study were not exactly the same as those included in either modified-Sharp XR method, correlations were high between the total MRI cartilage score in the wrist and total XR-JSN score in the same wrist (0.61 to 0.74) or in both hands, wrists, and feet (0.68 to 0.78). A subsequent study by Ostergaard *et al. *[10] similarly found strong correlation between a similar 5-point MRI-JSN scale and the van der Heijde-modified Sharp XR-JSN scale in the same hand and wrist.

## Conclusions

When MRI and XR images were scored for JSN in the same 13 joint locations that are typically scored in XR clinical trials of RA, the results agreed very well, with no statistically significant difference. Additional XR JSN scores in the feet and in the hand/wrist not imaged with MRI did not increase the number of JSN-positive patients, whereas the additional joints scored by using MRI increased involved joints and JSN-positive patient assignment. This suggests that MRI-JSN scoring may offer a viable alternative to XR-JSN scoring in multicenter clinical trials of RA. Replacement of XR with MRI in clinical studies of RA would allow longitudinal study designs to take advantage of the MRI improved ability to visualize erosion progression. The MRI relative longitudinal sensitivity to change and its ability to discriminate therapeutic effects specifically on JSN must be evaluated directly in coming studies.

## Abbreviations

CMC: carpometacarpal; ICC: intraclass correlation coefficient; JSN: joint-space narrowing; MCP: metacarpophalangeal; MRI: magnetic resonance imaging; PIP: proximal interphalangeal; RA: rheumatoid arthritis; RANKL: receptor activator of NF-κB ligand; ROC: receiver operating characteristic; T: Tesla; T_1_: longitudinal magnetic spin relaxation time; XR: X-ray.

## Competing interests

CGP is a shareholder in Spire Sciences, and a consultant for Abbott, Amgen, AstraZeneca, Biogen-Idec, Bristol Myers-Squibb, BioClinica, Celgene, Centocor, Core Lab Partners, Crescendo, Eli Lilly and Company, Genentech, Genzyme, Merck, Icon Medical Imaging, Novartis, Perceptive Informatics, Pfizer, Rigel, Roche, UCB, Wyeth, an employee of Spire Sciences, and a past employee of Synarc. JCDiC is a consultant for Abbott, Amgen, AstraZeneca, Biogen-Idec, Bristol Myers-Squibb, BioClinica, Celgene, Centocor, Core Lab Partners, Crescendo, Eli Lilly and Company, Genentech, Genzyme, Merck, Icon Medical Imaging, Novartis, Perceptive Informatics, Pfizer, Rigel, Roche, UCB, and Wyeth, an employee of Spire Sciences, and a past employee of Synarc. EO received grant/research support from Genentech and is a consultant for Genentech, and a member of the Speakers Bureau of Genentech. M-AB is an employee of Roche. AG is an employee of Roche. NG received Grant/Research Support from Genentech, is a consultant for Genentech, and is a member of the Speakers Bureau of Genentech.

## Authors' contributions

CP participated in protocol and experimental design, radiologic analysis, and drafted the manuscript. JD participated in protocol and experimental design, imaging acquisition and analysis of the reading results, and helped draft the manuscript. EO participated in protocol design, imaging acquisition, and reading results analysis. M-AB participated in protocol design, imaging acquisition, and reading results analysis. AG participated in protocol design, imaging acquisition, and reading results analysis. NG participated in protocol design, imaging acquisition, and reading results analysis. All authors read and approved the final manuscript.
